# Literary and Journalistic Notes

**Published:** 1904-03

**Authors:** 


### LITERARY AND JOURNALISTIC NOTES.

MARIANI'S COCA LEAF, Volume II., No. 5, January, 1904, contains many hints and suggestions relating to the use of coca and its preparations. It is a handsomely printed and well edited little periodical that will be sent on application to those who have not received it. Address, Mariani & Co., 52 West 15th Street, New York.

---

THE *Medical Library and Historical Journal* has entered upon its second volume. It is the only magazine of its kind published in the English language, its object being to promote interest in medical libraries and their growth. It is the official organ of the International Association of Medical Librarians and will publish the transactions of this society as well as those of the leading medical historical societies. The journal is printed on a fine quality of paper, is freely illustrated, and each number contains from 80 to 100 pages. It is published quarterly and the subscription price is $2.00 a year. The address of the editor is 1313 Bedford Avenue, Brooklyn.

---

The *Daily Medical* made its first appearance Monday, February 8, 1904, as an eight-page newspaper. It has regularly appeared since that date, Sundays excepted, publishing four pages each issue. It contains telegraphic news, original communications, new inventions recently patented, and editorial matter, together with a few advertisements. The subscription price is $1.00 a year. Address Dr. M. W. Curran, Managing Editor, 154 East 72d Street, New York.

---

The *Medical Book News,* published by P. Blakiston's Son & Co., Philadelphia, entered upon its second volume in January, 1904. It continues to give interesting information upon recent medical publications and is a valuable aid to the literary editor of a medical magazine.

---

Scientific bibliography.—J. B. Bailliere & Son, Hautefeuille St., No. 19, Paris, will soon publish a general catalogue of scientific works, comprising a detailed list arranged alphabetically, of names of authors embracing about five thousand works on medicine, natural history, agriculture, veterinary science, physics, chemistry, technology, and industry, with the date of publication, form, number of pages, figures and plates. An index of 17 pages, giving the names of authors of about 1,500 papers on scientific subjects is added. This indispensable bibliography of all the investigators will be sent to all readers of this Journal free of charge by writing Messrs. J. B. Bailliere & Son. A paid return postal card is sufficient.

---

The *International Medical Magazine,* originally a Philadelphia enterprise but lately published in New York, has been discontinued. The publishers, Messrs. E. B. Treat & Company, while regretting the discontinuance of the magazine, invite the friends of that publication to transfer their interest to the columns of the *Archives of Pediatrics,* and thus extend its field of usefulness.

THE *Maryland Medical Journal* announces that its February number which was destroyed in the great fire that swept over the business portion of that city, February 7 and 8, 1904, is being republished in Philadelphia, and will shortly appear. This was to be the Tuberculosis Exposition number, containing papers by Knopf, Flick, Adami, Ravenel, Thayer, Hoffman, Salmon, and others. We regret the calamity that befell our distinguished contemporary, but are glad it will be enabled to carry out its original purpose in regard to its February edition.

---

THE *Interstate Medical Journal* for January, 1904, appeared in what it is pleased to call, an Annual Progress Number. It contained reviews of the several branches of medicine, editorial comment and book reviews.

---

THE fourth annual report of the work of the cancer laboratory of the New York State Board of Health, conducted at the Gratwick Research Laboratory, University of Buffalo, for a period beginning in 1902 and ending in 1903, was transmitted to the legislature February 1, 1903. It was sent out in the month of December of the latter year. It contains the report of the director, Dr. Roswell Park, and several scientific papers prepared by Drs. Harvey R. Gaylord, Gary N. Calkins, G. H. A. Clowes, Herman G. Matzinger, Irving Phillips Lyon and Roswell Park. These all pertain to investigations bearing on the question of cancer. Many of the papers are illustrated with microscopic sections. It is the most complete exhibition of research work that has been published in this country relating to the origin, causes and nature of cancer. It will, undoubtedly, attract the attention of laboratory workers all over the world and reflects the highest credit upon all concerned in its preparation.

